# The patent dispute over the breakthrough mRNA technology

**DOI:** 10.3389/fbioe.2022.1049873

**Published:** 2022-11-03

**Authors:** Guillermo Aquino-Jarquin

**Affiliations:** Laboratorio de Investigación en Genómica, Genética y Bioinformática. Torre de Hemato-Oncología, 4to Piso, Sección 2. Hospital Infantil de México Federico Gómez, Ciudad de México, Mexico

**Keywords:** mRNA-LNP, Moderna, Pfizer/BioNtech, COVID-19 vaccine, Patent infringement, Lawsuit

## Introduction

The development and approval of effective COVID-19 vaccines represented a significant advance during the ongoing pandemic (Szabo et al., 2022). Numerous vaccine candidates are based on the lipid nanoparticle-encapsulated nucleoside-modified mRNA (mRNA-LNP) platform. However, Moderna/US NIAID (mRNA-1273) and Pfizer/BioNTech (BNT162b2 or Comirnaty^®^) were the first mRNA-LNP vaccines to enter clinical trials and receive emergency authorization and regulatory approvals (Pfizer-LOA-051021, 2021; Moderna-LOA-08312022, 2022).

On 26 August 2022, Moderna filed a patent infringement lawsuit against Pfizer and BioNTech in the United States District Court for the District of Massachusetts and the Regional Court of Düsseldorf in Germany (Complaint for Patent Infringement, 2022). Moderna’s lawsuit involves three patents that claim priority to applications filed between 2011 and 2016 covering its foundational intellectual property (Moderna Press Release, 2022). Interestingly, Moderna seems to have evidence that Pfizer and BioNTech unlawfully copied the mRNA chemical modification, particularly N1-methylpseudouridine (m1*ψ*) — to enhance immune evasion and keep protein production—, and the design of encoding complete spike protein. Both represent two critical components of their patented mRNA technology platform (Moderna Press Release, 2022).

Nevertheless, in the first instance, there are several similarities between the vaccine developed by both biotech, among which stand: 1) both formulations use the same mRNA technology encoding for the Spike protein of SARS-CoV-2, 2) both mRNA are encapsulated in lipid nanoparticles, 3) the route of administration is intramuscular, and two doses are required in each case, 4) both showed the most excellent efficacy in preventing symptomatic COVID-19 disease in clinical trials, with efficacies reaching 95% (BNT162b2) and 94.5% (mRNA-1273), 5) the side effects that both vaccines have presented are very mild. Such similarities have been pointed out by Moderna.

However, there is no evidence that both vaccine manufacturers employ an additional adjuvant that amplifies the immune response with their vaccines and becomes more potent. BioNTech/Pfizer has indicated that RNA acts as an adjuvant (Chung et al., 2020). However, the LNP carrier may act as an adjuvant since other lipids have been reported to have adjuvant properties (Perrie et al., 2016). In this regard, the Pfizer/BioNTech vaccine is somewhat more potent than Moderna’s [e.g., evoking vigorous production of antibodies specific for the receptor-binding domain (RBD), T cell repertoire, and favorable cytokine responses] (Chung et al., 2020), which could be a consequence of the subtle differences that the Pfizer/BioNTech vaccine possesses.

## Critical comparison of the COVID-19 mRNA vaccines made by Moderna and Pfizer/BioNTech

To analyze the basis for Moderna’s assertions that Pfizer has infringed their patents, a head-to-head comparison between both mRNA vaccines needs to be carried out. Initially, it is not easy because most of the structural components of Moderna’s vaccines (mRNA-1273, mRNA-1273.211, and mRNA-1273.351) have not been fully disclosed.

However, through a detailed dissection of mRNA structural components, it will be possible to know if there are critical dissimilarities or not among both mRNA-LNP formulations. In this regard, the latter could determine whether Pfizer/BioNTech’s mRNA possesses an “inventive step” regarding Moderna’s technology platform or if their invention results ‘obvious’ for a “person of ordinary skill in the art” (e.g., an average molecular biologist) (Sherkow, 2017). Hence, if such changes in the structural components lack an “inventive step” over that Moderna patented previously, the legitimacy of the Pfizer/BioNTech vaccine could be questioned.

The landscape is not entirely clear, and it depends on the glass with your look. For example, focusing on the mRNA 5’ untranslated regions (5’-UTR), Moderna has not revealed the 5’-UTR used in their vaccine. Nevertheless, what is known is that Moderna implemented the incorporation of m*Ψ* modified nucleotides into the mRNA-1273. Concerning this, Pfizer/BioNTech incorporated in the BNT162b2 mRNA a 35-nt sequence (*Ψ*C*ΨΨ*C*Ψ*GG*Ψ*CCC​CAC​AGA​C*Ψ*CAG​AGA​GAA​CCC​GCC) derived from the 5’-UTR of the highly expressed human gene α-globin (Granados-Riveron and Aquino-Jarquin, 2021; Xia, 2021). Even though the sequence of this 5’-UTR might not be the same as that used by Moderna, BNT162b2 mRNA has m1*ψ*. Therefore, attorneys would have to examine whether the incorporation of m*Ψ* modified nucleotides is “obvious,” regardless of the positions in the 5’-UTR sequence where the modifications are incorporated, to enhance prolonged stability and increase protein production (as initially claimed by Moderna).

On the other hand, it is remarkable that the BNT162b2 vaccine produces about 3.3 times as many Spike proteins as Moderna’s mRNA-1273 vaccine (Xia, 2021). The latter would explain why the doses of BNT162b2 are lower (30 μg) than the amounts of mRNA-1273 (100 μg). Again, the examiners would have to resolve whether decreasing the vaccine mRNA (μg) is due to increasing the mRNA translation efficiency, as a result of the m1*ψ* incorporation in the mRNA sequence (including the 5’-UTR), or simply this is because BNT162b2 uses a fragment of 35-nt from 5’-UTR of the human α-globin, which is already known to be highly expressed. The latter could be understood if Pfizer/BioNTech demonstrates that incorporating native nucleotides in its mRNA vaccine leads to the same results as using m1*ψ*, regardless of which 5’-UTR region is used. Suppose they achieve the same results in terms of mRNA translation efficiency. In that case, Pfizer/BioNTech could appeal that they have obtained an “improvement” on Moderna’s invention, perhaps of less technical complexity, where the patent infringement may not be pointed out.

In such cases, the court could review the lab notebooks used during the research and development of each vaccine to identify the kind and the length of the UTR sequences, chemically modified nucleotide incorporated, and lipid nanoparticle formulations, among other details that Moderna believes were plagiarized from their mRNA platform.

## The coronavirus mRNA vaccine technology is at the center of a patent battle

Before the COVID-19 pandemic, government researchers at the U.S. National Institutes of Health (NIH) and Moderna collaborated on developing vaccines for other coronaviruses (Ledford, 2021).

When the SARS-CoV-2 outbreak was imminent, Moderna and NIH collaborated on developing a functional vaccine for COVID-19. However, a U.S. patent application was filed by Moderna, with no NIH scientists included as inventors. In this matter, Moderna has indicated that no NIH scientists designed the vaccine claimed in the U.S. patent application. For its part, NIH has commented that it believes three federal scientists should be included in the patent application as co-inventors with the Moderna scientists. Nevertheless, there is no evidence supporting that both instances are in litigation for this fact (Mantooth, 2021).

This year, several patent lawsuits have been filed over the LNP technology employed in Pfizer/BioNTech and Moderna’s COVID-19 vaccines. On 10 January 2022, Pfizer claimed that it entered into an agreement with Acuitas Therapeutics for a lipid nanoparticle delivery system for mRNA vaccines and therapeutics (Pfizer Press Release, 2022). However, on 28 February 2022, Arbutus Biopharma and Genevant Sciences, two biotech companies specializing in developing lipid nanoparticles, claimed to own the intellectual property of nanoparticle lipids used by Moderna for COVID-19 vaccines. As a result, Arbutus and Genevant have recently sued Moderna in the U.S. District Court for the District of Delaware for allegedly infringing their nanoparticle formulations (Shores, 2022). On 17 March 2022, Alnylam Pharmaceuticals, an American biopharmaceutical company focused on the discovery, development, and commercialization of RNA interference therapeutics, also sued Moderna and Pfizer (separately) in the U.S. District Court for the District of Delaware, alleging that both infringed its patent concerning one of the four lipid components encapsulating an mRNA payload in commercialized lipid nanoparticles (Brittain, 2022; Shores, 2022).

Furthermore, the German company CureVac, an early mRNA pioneer whose mRNA vaccine stalled after showing just 47% efficacy against COVID-19 in a late-stage trial (2022), protects several inventions that the company considers critical to the design and development of BioNTech’s mRNA COVID-19 vaccine. On 7 July 2022, CureVac filed suit against BioNTech in the German Regional Court, seeking compensation for infringement of four CureVac patent claims concerning the features of the mRNA payload and lipid formulation used to make the BioNTech coronavirus vaccine (CureVac Press Release, 2022; Shores, 2022). On 12 July 2022, Alnylam again separately sued Moderna and Pfizer/BioNTech, but this time, the lawsuit was for the entire LNP (Shores, 2022). Thus, Moderna is not exempt from lawsuits and would also have to resolve the dispute over whether or not they infringed the patents regarding the same LNP delivery technology. So “whoever is without guilt cast the first stone.”

## Looking ahead

In drug and vaccine development, inventors regularly file multiple patents to cover different aspects of a single invention (Ledford, 2021). In the “Nature of the Case” (Complaint for Patent Infringement, 2022), Moderna claims that although Pfizer and BioNTech initially considered different vaccine designs, they ultimately chose to use the same structure of mRNA encoding for the full-length spike protein of SARS-CoV-2 (Moderna Press Release, 2022).

While encoding the full-length spike seems to be a prominent option for the vaccine, critical questions arise: what evidence does Moderna have to infer or accredit that Pfizer/BioNTech’s vaccine design is precisely the same? The design and preclinical testing of nucleoside-modified mRNA vaccines are based on years of research. Thus, based on what did Pfizer/BioNTech reach its first mRNA-based COVID-19 vaccine in just 1 year?

The above may result from compressed development times in the face of the enormous threat from the virus. For example, once the safety pitfalls were overcome, the Pfizer/BioNTech vaccine was more easily adapted to platform manufacturing technologies and supply chains, representing the fastest pathway for its availability (Graham, 2020).

However, although the rationale for mRNA technology is relatively simple, it is clear that researchers have had to work for years developing technologies to allow mRNA to work inside our cells and produce proteins for evoking an immune response that protects us against diseases. For instance, Pieter Rutter Cullis’s research, best known for developing ionizable cationic lipids, helped develop lipid nanoparticles as systems to deliver therapies and vaccines, a critical enabler for mRNA technology (Pfizer News Media, 2022). Furthermore, the work of Katalin Karikó and Drew Weissman at the University of Pennsylvania was able to engineer the chemistry of mRNA in a way that could get into cells avoiding autoimmunity and without interfering with desired immune responses (Pfizer News Media, 2022). Thus, these breakthrough findings undoubtedly led to the development of the first mRNA vaccines for COVID-19 and confirmed the promise of the mRNA technology.

The key also seems to be in partnering and collaboration through various licensing and strategic research collaborations that Pfizer/BioNTech may have established to develop mRNA-based vaccines (e.g., Acuitas Therapeutics). Indeed, Moderna and BioNTech have obtained licenses from Karikó and Weissman’s patent (Shores, 2022).

For now, the patent-holders only seek monetary damages (“reasonable royalty”) rather than an injunction against any defendants (CureVac Press Release, 2022; Moderna Press Release, 2022). For example, Moderna is seeking damages for revenue from Pfizer & BioNTech derived from sales in the United States and its domestic manufacture for supply outside of 92 low- and middle-income economies eligible for access through COVID-19 Vaccines Global Access (COVAX) (Fulker, 2020; Mast, 2022). Furthermore, Moderna has stated that it does not pretend Pfizer/BioNTech vaccine removed from the market, nor is it seeking an order preventing future sales, so the lawsuit will not affect the expected rollout of reformulated boosters in the short term (Mast, 2022; Moderna Press Release, 2022). However, it is clear that by declaring itself a pioneer of the mRNA technology platform, Moderna is seeking to expand its mRNA technology into new vaccines for infectious diseases and treatments for cancer, various autoimmune disorders, and rare conditions for the lucrative that this implies.

This breakthrough biopharma technology has triggered a set of patent lawsuits that are in their early stages. For its part, Pfizer/BioNTech asserted that its work is original and will vigorously defend it against all allegations of patent infringement (Pfizer Press Release, 2022). However, the patents’ validity of one o more LNP formulations can be invalidated or canceled through Inter Partes Review (IPR)/post-grant review (PGR) proceedings (Shores, 2022), and all patents can be subject to court scrutiny. Therefore, those Biotechs that could be involved probably have to amend their claims and hence their complaints based on patent decisions issued by the court. In this sense, if it were the case that any of the first LNP formulation patents are invalidated or canceled, this could facilitate utilizing a plethora of delivery formulations without licensing involved with party patent-holders. In contrast, if such patent-holders win the lawsuit, the technology covered by the patents may be subject to an application for licensing to be able to use them. This would also depend on whether the patent survives after invalidity challenges (Shores, 2022).

Finally, the dispute over who deserves to be credited for patents concerning mRNA technology seems more complex than previously thought, and the patent decisions involved could take several years. However, patent counsels must set effective strategies to reach additional agreements for utilizing the range of delivery formulations, mRNA platforms, and RNA manufacturing processes for the next generation of mRNA vaccines.
